# Functional Cell Types in Taste Buds Have Distinct Longevities

**DOI:** 10.1371/journal.pone.0053399

**Published:** 2013-01-08

**Authors:** Isabel Perea-Martinez, Takatoshi Nagai, Nirupa Chaudhari

**Affiliations:** 1 Department of Physiology and Biophysics, University of Miami Miller School of Medicine, Miami, Florida, United States of America; 2 Department of Biology, Keio University School of Medicine, Yokohama, Japan; 3 Program in Neurosciences, University of Miami Miller School of Medicine, Miami, Florida, United States of America; German Institute of Human Nutrition Potsdam-Rehbruecke, Germany

## Abstract

Taste buds are clusters of polarized sensory cells embedded in stratified oral epithelium. In adult mammals, taste buds turn over continuously and are replenished through the birth of new cells in the basal layer of the surrounding non-sensory epithelium. The half-life of cells in mammalian taste buds has been estimated as 8–12 days on average. Yet, earlier studies did not address whether the now well-defined functional taste bud cell types all exhibit the same lifetime. We employed a recently developed thymidine analog, 5-ethynil-2′-deoxyuridine (EdU) to re-evaluate the incorporation of newly born cells into circumvallate taste buds of adult mice. By combining EdU-labeling with immunostaining for selected markers, we tracked the differentiation and lifespan of the constituent cell types of taste buds. EdU was primarily incorporated into basal extragemmal cells, the principal source for replenishing taste bud cells. Undifferentiated EdU-labeled cells began migrating into circumvallate taste buds within 1 day of their birth. Type II (Receptor) taste cells began to differentiate from EdU-labeled precursors beginning 2 days after birth and then were eliminated with a half-life of 8 days. Type III (Presynaptic) taste cells began differentiating after a delay of 3 days after EdU-labeling, and they survived much longer, with a half-life of 22 days. We also scored taste bud cells that belong to neither Type II nor Type III, a heterogeneous group that includes mostly Type I cells, and also undifferentiated or immature cells. A non-linear decay fit described these cells as two sub-populations with half-lives of 8 and 24 days respectively. Our data suggest that many post-mitotic cells may remain quiescent within taste buds before differentiating into mature taste cells. A small number of slow-cycling cells may also exist within the perimeter of the taste bud. Based on their incidence, we hypothesize that these may be progenitors for Type III cells.

## Introduction

Taste buds are aggregates of 50–100 specialized sensory cells embedded in the stratified oral epithelium. Taste bud cells have characteristics of both epithelial cells and neurons insofar as these cells are a renewing epithelium and, at the same time, are excitable sensory receptors that communicate synaptically to neurons.

Taste bud cells exhibit a range of cell shapes and dimensions as reported in early electron microscopic studies [1]. Cells in taste buds are specialized; each cell detects at most, a subset of compounds that are structurally related or generate a common sensory submodality (e.g. sweet). In keeping with these specializations, the three currently recognized types of taste bud cells exhibit very distinct morphological features, transcriptomes and cellular functions. Recent well-coordinated analyses of expression of marker mRNAs or proteins with cellular function have begun to reveal the logic underlying the organization and function of taste buds [2]. Specifically, Type I cells are termed “glial-like” because they appear to function in clearing neurotransmitters [3], ensheath other taste bud cells with lamellar processes [4] and may regulate the ionic milieu [4], [5]. Type II (Receptor) cells express G-protein-coupled receptors (GPCR) selective for sweet, bitter or umami tastants and downstream effectors that mediate inositide-mediated Ca^2+^ signaling [6]–[8]. Type III cells are the most neuron-like cells: they possess specialized chemical synapses, synaptic vesicles, voltage-gated Ca channels and several other neuronal proteins [9], [10].

Like other epithelial cells, individual taste bud cells have a limited life span and are part of a renewing population. Throughout the life of the animal, taste cells are continuously replaced via cell proliferation along the basement membrane of the epithelium. Electron microscopic studies found that ^3^H-thymidine is first incorporated into basal epithelial cells outside taste bud boundaries and only appears within taste buds with the passage of time [11], [12]. This suggested that cells are born in the basal epithelium adjacent to taste buds and migrate in to replenish taste buds. More recent studies using genetic tools have shown clearly that adult taste buds are derived from, and renewed by proliferation in local epithelium during embryonic development, early postnatal growth, and in the adult [13], [14]. Further, there exist progenitor cells in the basal epithelium that give rise to both taste buds and the surrounding nonsensory epithelium [15].

Early estimates using ^3^H-thymidine suggested that the average lifespan of taste bud cells in rodents is 8–12 days [11], [12]. Farbman [16] suggested that different morphological classes of cells may turnover at rather different rates, with certain cells being particularly long lasting. More recent studies utilizing BrdU-labeling also suggested that cellular lifespans within the taste bud may be heterogeneous [17]. However, the identities of the slow- and fast-cycling cells were not addressed, and it has been an open question whether Types I, II, and III taste bud cells have similar lifespans.

In the present study, we have used a newly developed nucleotide analog, 5-ethynil-2′-deoxyuridine (EdU), to label and detect proliferating cells with higher specificity and sensitivity than is possible with earlier probes such as BrdU. Because the signal for EdU is exceptionally strong, we have been able to combine EdU incorporation with multi-color immunofluorescent identification of circumvallate taste cell types. All three classes of taste cells display a distinctive time course of EdU labeling. Importantly, we show that the most neuron-like of taste cells, the Type III cells, turn over less frequently and appear to be very much longer lived than the other cells in taste buds.

## Materials and Methods

### Ethics Statement

Mice were handled and euthanized according to NIH Guidelines for the Care and Use of Laboratory Animals; procedures were approved by the University of Miami Institutional Animal Care and Use Committee.

### Animals

Adult male and female mice were housed in a 12 h light/dark cycle (5∶30am to 5∶30pm). All mice were of the *PLCb2*-GFP transgenic strain [18] to facilitate identifying Type II (Receptor) taste cells, as described below. Mice were killed by CO_2_ inhalation followed by cervical dislocation before dissecting out tissues.

### EdU Detection through Successive Mitoses

**Figure 1:**
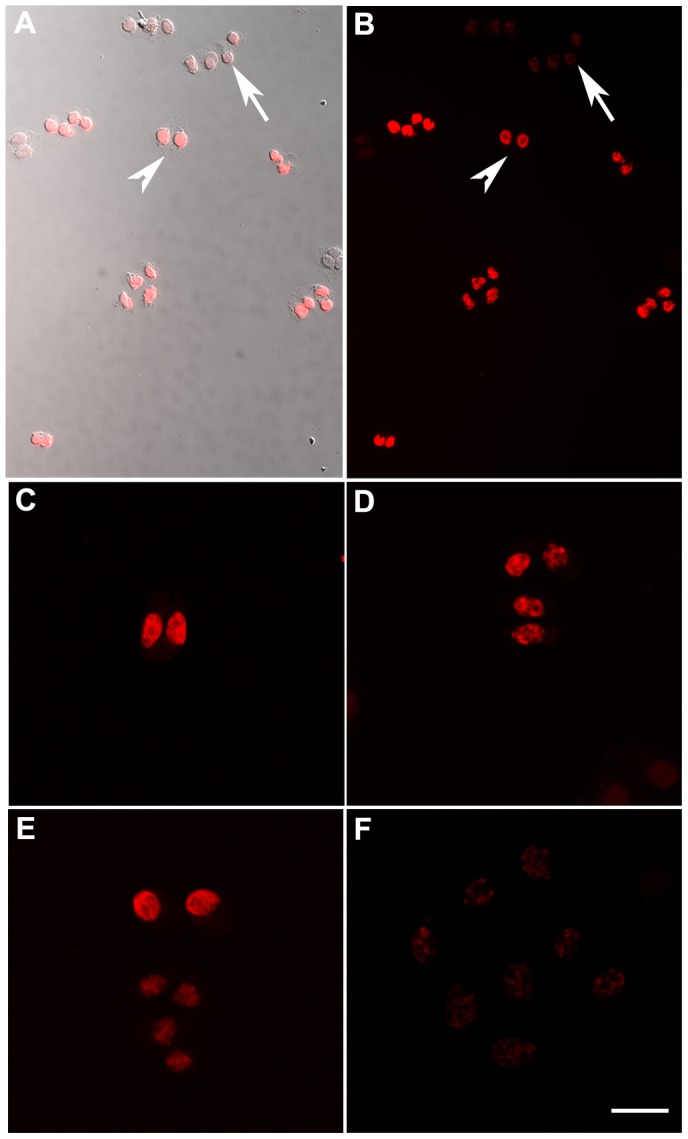
Estimating the efficacy of EdU detection through successive mitoses. CHO cells plated at clonal density were exposed to a 4 h pulse of EdU (10 µM) followed by 24 h or 48 h chase in medium lacking EdU. Cultures were fixed and stained for EdU and well-isolated clones were imaged. **A, B,** EdU-labeled cells (arrowhead) are brightly fluorescent and distinguished from unlabeled cells (arrow). **C, D,** Fluorescence in 2- and 4-cell clones is well above background. **E,** In this clone, asynchronous cell divisions produced bright fluorescence in top two cells after two mitoses, and less bright fluorescence in the lower four cells after three mitoses. **F,** After three mitoses, fluorescence is barely above background. Scale, 20 µm.

We first evaluated how many successive mitoses EdU-labeled cells can undergo before EdU becomes undetectable. We exposed actively dividing CHO cells to a 4 h pulse of EdU (10 µM; Invitrogen), then returned cells to normal growth medium, and fixed cultures at different times. After the click-reaction (see below), the EdU signal was readily distinguished from background (Figure 1A,B). Brightly fluorescent nuclei were visible in clones of 2 or 4 cells (Figure 1 C,D) that is, following 2 mitoses. Clones that had undergone upto 3 mitoses post-labeling had EdU fluorescence significantly above background while clones resulting from 4 or more mitoses were not consistently distinguished from background fluorescence (Figure 1 E,F). Using Image J software, we quantified fluorescence intensity in 245 cells in clones. All cells within a clone exhibited similar fluorescence, and its intensity correlated with the number of cells in the clone.

*In vivo* labeling with EdU is likely much less efficient than in cell culture. Unlike our result with CHO cells, *in vivo* labeled tissues did not yield a consistent relationship between fluorescence intensity and post-labeling interval. Nevertheless, based on the brightness of EdU+ nuclei in tissue sections, we believe that EdU should be detectable through at least two sequential asymmetric mitoses in epithelium.

### EdU Labeling *in vivo*

In preliminary trials, we intraperitoneally (i.p.) injected mice with either 100 µg or 200 µg (i.e. ≈5 mg or 10 mg/Kg) EdU in saline, and evaluated tissues 2–6 days later. Both doses yielded similar numbers and fluorescence intensity of labeled cells in lingual epithelium and in taste buds. Hence, subsequent EdU injections that yielded data throughout the present study were performed with a single dose of 100 µg/mouse. There is a peak of DNA synthesis in lingual epithelium during the dark phase of the diurnal cycle [16]. Hence, we carried out EdU injections at 7p.m. Mice were returned to their home cages, and were killed 4 hours to 40 days after the injection. The earliest time point, 4 h, was used to estimate the number of cells that initially incorporated EdU.

One hour prior to sacrifice, we injected mice with 16 mg 5-hydroxy tryptophan (Sigma Chemical Co.), a precursor of 5HT. This optimizes immunostaining for serotonin, a reliable marker of Type III cells [10], [19]. After euthanasia, circumvallate taste papillae were dissected and immersion fixed for 1 h in 4% paraformaldehyde in Phosphate Buffered Saline (PBS; in mM: 154 NaCl, 1 KH_2_PO_4_, 3 Na_2_HPO_4_, pH7.4). After cryoprotection, 25 µm floating cryosections were permeabilized (15 min in 0.25% Triton X-100 in PBS) and non-specific binding was blocked (15 min in 1% bovine serum albumin). EdU was then visualized by reacting the alkyne group in the dark for 30 min to 5 µM Alexa 594-conjugated azide (Invitrogen) via click chemistry [20]. Sections were then washed in 1% BSA in PBS for 10 min before proceeding to immunofluorescence.

### Immunofluorescence Microscopy

Reaction conditions during the alkyne-azide click reaction destroy GFP fluorescence although the antigenicity of GFP persists. Hence, we used immunofluorescence to detect GFP in taste buds from *PLCb2*-GFP mice, allowing us to identify Type II taste bud cells. We also used antibodies against KCNQ1, a plasma membrane ion channel found in all taste bud cells [21] to define the limit of each taste bud. To detect Type III cells, we selected 5-HT, an amine that is reliably localized by electron microscopy to the cytoplasm of all the synapse-bearing (i.e. Type III) cells [10]. After the click reaction to visualize EdU, tissue sections were processed for immunofluorescence as we described previously [5]. Sections were incubated overnight at room temperature in a mixture of three primary antibodies: chicken anti-GFP (1∶1000; GFP-1020, Aves Labs), goat anti-KCNQ1 (1∶1000; sc-10646; Santa Cruz Biotechnology) and goat anti-5HT (1∶2000; S5545, Sigma Immunochemicals). After thorough washing in PBS, sections were incubated in a mixture of fluorescent secondary antibodies: donkey anti-chicken Dylight 405 (1∶1000; 703-475-155; Jackson Labs), donkey anti-rabbit Alexa Fluor 488(1∶1000; A21206, Invitrogen ) and donkey anti-goat Alexa Fluor 647 (1∶1000; A21447, Invitrogen) at room temperature for 2 hours.

**Figure 2:**
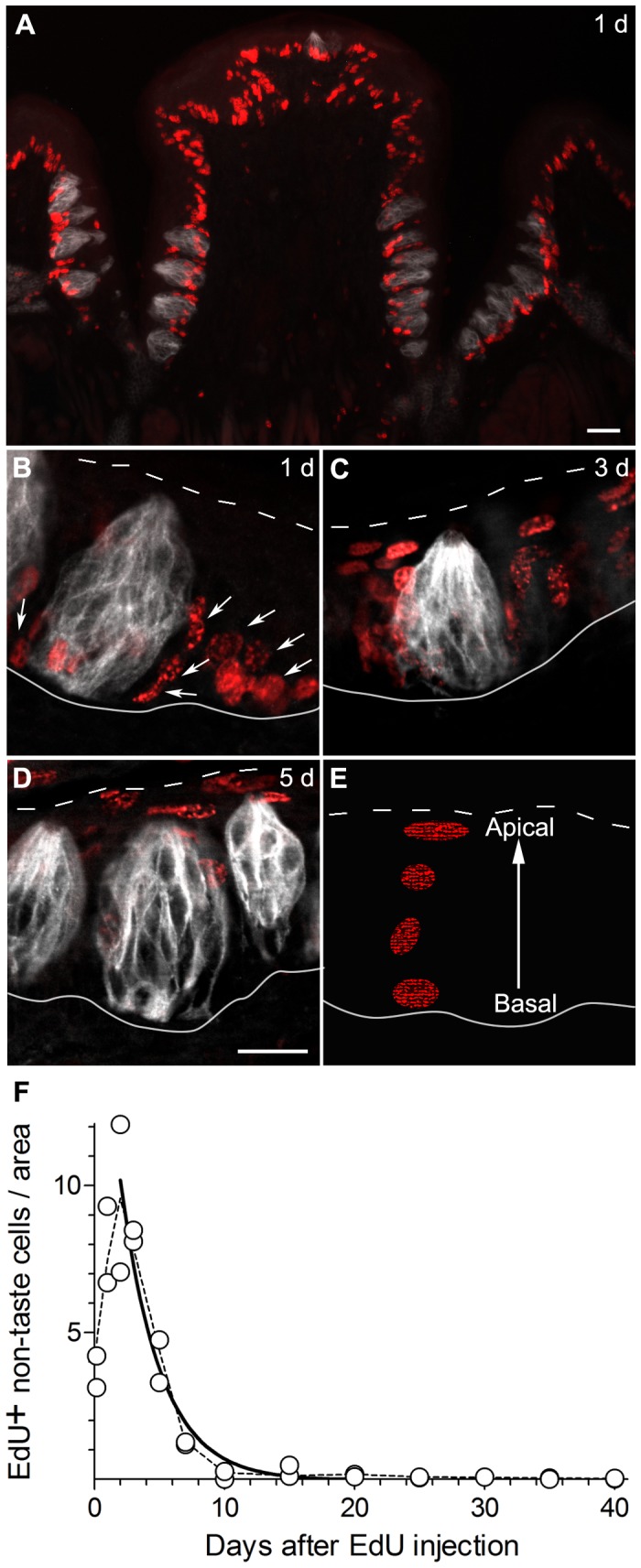
EdU-labeled nuclei in non-taste lingual epithelium surrounding taste buds turn over rapidly. A, wide-field fluorescence micrograph of circumvallate papilla, 1 day after a single pulse of EdU. Many EdU-labeled nuclei (red) are visible along the base of the epithelium. Taste buds are immunostained (grey) for KCNQ1, a marker for all taste cells. The location of EdU+ nuclei, whether inside or outside of taste buds cannot be judged from a wide-field micrograph such as this. **B–D**, Higher power single plane confocal micrographs of circumvallate taste buds, 1, 3, or 5 days after injecting EdU. The large majority of EdU-labeled epithelial cells are outside of taste buds. Basal and apical limits of the epithelium are indicated with white solid and dotted lines respectively to highlight the rapid migration of newly-born non-taste cells from the stratum basale to the superficial epithelial strata. **E**, Cartoon depicting progression of EdU-labeled non-taste nuclei vertically through the epithelium and their simulataneous change of shape from ovoid at the base to horizontally flattened at the apical surface. **F**, Plot of EdU labeled non-taste nuclei per unit area *versus* days post-injection. Data were obtained from micrographs of circumvallate trench such as those in **B–D**. Each symbol represents data from a separate mouse. The solid line is a best-fit 1-phase exponential decay curve that yields a half-life of 2 days (R^2^ = 0.98) for non-taste epithelial cells surrounding taste buds. The dashed line is a smoothed line through the average values at each time point in the time course. Scale bars, 20 µm.

Images were captured with a 20x water-immersion objective on an Olympus FV1000 laser scanning confocal microscope. To avoid optical bleed-through when imaging at four wavelengths, laser scanning was conducted in two phases (405, 594 and 647 laser lines simultaneously and 488 laser separately). Optical sectioning was to ≈1.5 µm thickness. Micrographs in Figures 1 and 2A (as indicated in figure legends) were captured on a wide-field Zeiss Axiophot microscope.

### Cell Counting and Data Analysis

Data from circumvallate papillae from a total of 42 mice are presented. In 3 additional mice, EdU incorporation was poor throughout the lingual epithelium, and in other tissues, presumably because of a sub-optimal site for the EdU i.p. injection. We did not include data from these mice. Circumvallate papillae were sectioned and stained as detailed above and all sections were mounted. Between 5 and 18 sections for each mouse were then confocally imaged as Z-stacks. Every taste bud in these Z-stacks was counted, and every EdU+ nucleus in the trench non-taste epithelium in the images was scored. For each mouse, 40 to >200 taste buds were analyzed, for a total of 4181 taste buds across the entire series. Double counting of the same taste bud was avoided as follows. First, our frozen sections (25 µm) are about the same thickness as circumvallate taste buds (20–40 µm). We counted only those taste buds that were included in more than 80% of the thickness of each cryosection. Second, we counted only a subset of the cryosections from each papilla and these sections were not sequential.

The number of all EdU+ taste bud cell nuclei in a given section was normalized to the number of taste buds in that section. We also counted all EdU+ nuclei in the non-taste epithelium of circumvallate trenches of each section, and normalized the count to the number of taste buds in that section (as a surrogate for epithelial area). In all graphs, each data point is derived from a separate mouse and represents EdU+ nuclei, normalized as above. The values of EdU+ nuclei per taste bud from each mouse (i.e. *not* the averages at each time point), were then fit with a non-linear exponential decay curve using Prism 5.0 (GraphPad Software).

To objectively quantify the loss of cells from each population, we applied two strategies. First, we iteratively repeated each of the non-linear analyses using each possible time (day 2, 5, 10 etc.) as a starting point for the fit. For each of the five data series, we selected the one curve that yielded the highest goodness of fit, R^2^, meaning that it best represents the data. Second, we limited the mathematical fit to the falling phase of each data series. We calculated the mean value from all animals at each given time-point. Where the data set displayed a broad plateau rather than a sharp peak, the last time-point of the plateau was set as the beginning of the falling phase. This is where cell loss exceeds inflow of new cells into the population. In all five data series, the curve that yielded the highest goodness of fit was also the one that was initiated at the latest peak/plateau point. Data for the non-taste cells were fit starting at day 2. The remaining data series were all fit starting at day 10.

All data points, whether or not they were included in the non-linear decay fit, are presented on the graphs. The best-fit values for half-life (in days) and the goodness of fit (R^2^) for each decay curve are given in the corresponding figure legends. Each graph also displays a smoothed line through the mean values at each time point to illustrate the closeness of the raw data to the calculated fit curve.

## Results

### Epithelial Cells Outside Taste Buds Turn Over Rapidly

Earlier studies showed that the bio-availability of i.p. injected BrdU in the mouse is 2–3 hours [22]. In mouse lingual epithelium, the duration of S-phase is ≈5 h while G2+ half of M phase is estimated at 4.2 h [23]. Assuming that EdU has similar dynamics to BrdU, very few labeled cells detected 4 h after injection should have already completed mitosis. Thus, a time-point 4 h after EdU injection provides an estimate of initial labeling. We first analyzed EdU incorporation in the non-taste lingual epithelium immediately surrounding circumvallate taste buds, from 4 h to 40 days post-injection. Large numbers of EdU+ cells were seen in the basal layer of epithelium within 1 day of the EdU injection (Figure 2A). Over the next two days, there was a nearly 3-fold increase in the number of EdU+ epithelial nuclei *outside* taste buds, relative to initial labeling (Figure 2F), suggesting that on average, initially labeled basal epithelial cells produce progeny through 2 successive asymmetric mitoses. Cells born in the basal layer of the epithelium (Figure 2B) migrated up through the height of the epithelium and were predominantly located near the mucosal surface by 5 days post-injection (Figure 2B–E). Figure 2F is a quantification of these data. After a peak on Day 2, the population of EdU-labeled non-taste epithelial cells declined rapidly with a half-life of about 2 days (R^2^ = 0.93).

Interestingly, a few labeled cells persisted in the basal layer of the epithelium after the main wave of EdU+ cells had exited the apical face (not shown). These are similar to the label-retaining or slow-cycling reserve progenitor cells observed in other regenerative epithelia such as the intestine and skin [24], [25]. On average, label-retaining cells represented about 4% of the proliferative basal layer at 20 days, gradually declining to 0.6% at 40 days.

### Most EdU Labeled Cells within Taste Buds Appear after a Delay

**Figure 3:**
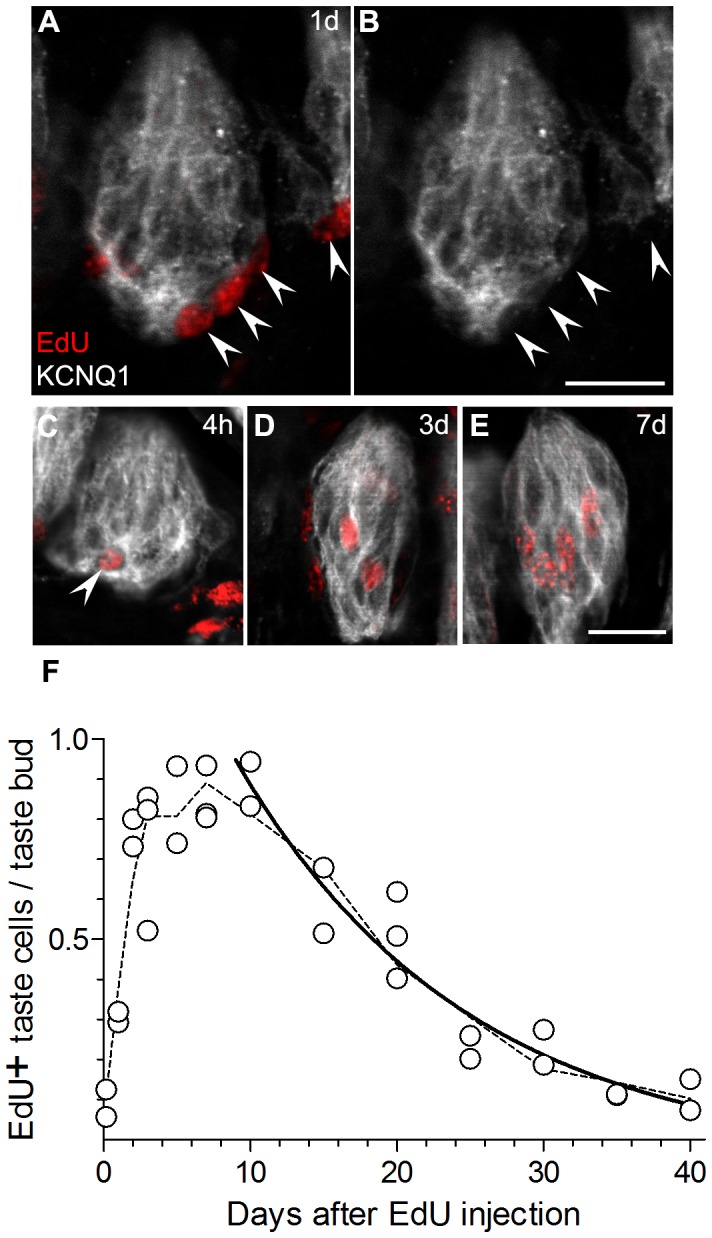
EdU-labeled nuclei appear in taste buds within 1 day of injection. Cryosections of circumvallate papillae were stained for EdU (red) and KCNQ1 (grey) as in Figure 2. All micrographs are confocal single (≈1.5 µm) optical sections. **A,** EdU+ nuclei are closely apposed to the base of the taste bud 1 day post-injection. **B,** The same field as in **A**, viewed for KCNQ1 alone reveals “dimples” where EdU+ nuclei (arrowheads) appear to push in without penetrating into the taste bud. **C,** Example of an EdU+ nucleus (arrowhead) located inside a taste bud 4 h after EdU injection. Optical section was selected passing through the widest, most brightly stained part of the nucleus. **D, E,** Representative high magnification views of taste buds from mice killed 3 days or 7 days after EdU injection. **F,** Aggregate data for EdU+ nuclei located within taste buds. Each symbol represents data from one mouse. Solid line is a 1-phase exponential decay curve that yields a half-life of 11 days for EdU+ taste cell nuclei (R^2^ = 0.92). A smoothed (dashed) line through averages at time points is also included. Scale bars, 20 µm.

Because large numbers of EdU-labeled cells are located very near taste buds, we used two sequential steps to verify whether a labeled cell was truly inside or outside a taste bud. First, we used KCNQ1-immunostaining to define the limits of taste buds precisely; this ion channel is expressed in most or all taste bud cells as reported [21] and we have confirmed. Second, we scored each EdU-labeled nucleus by viewing 2-color fluorescence (for EdU and KCNQ1) through successive ≈1.5 µm thick optical sections in a Z-stack. We found this analysis essential because many nuclei labeled within 1 day of injection are closely apposed to the base of a taste bud. In successive images of a Z-stack, these labeled nuclei appear to push into the face of the taste bud without penetrating it. Such “dimples” or indentations produced by extragemmal cells are apparent on the basal face of most taste buds (Figure 3A,B). Most EdU+ nuclei apposed to the base of taste buds tend to display a broad oval aspect face-on (Figure 3A), and are slightly flattened when viewed in profile. Throughout this study, we identified EdU+ nuclei as *within* taste buds only if KCNQ1-immunostaining was detected completely surrounding the labeled nucleus in all Z-stack images containing that nucleus (see Figure 3C–E).

Using the stringent criteria described here, and contrary to our expectations, we observed a few labeled cells within taste buds as early as 4 h post-injection (20 cells in 226 taste buds). These labeled cells, constituting >0.1 cell per taste bud (for one EdU pulse), could represent at least two distinct possibilities. First, a few labeled cells from the surrounding non-sensory epithelium may rapidly migrate into the taste bud prior to undergoing mitosis. Alternatively, the intragemmal label may represent taste bud-resident cells that are mitotically active. Our current data do not allow to distinguish between these possibilities, although the second seems more plausible.

By 5 days post-injection, the number of intragemmal EdU-labeled cells increased to ≈1.0/taste bud. The 10-fold increase in the number of labeled cells in taste buds is likely due to influx of cells from the surrounding undifferentiated basal epithelium. Labeled cells within taste buds exhibited a broad peak of labeling 5–7 days post-injection (Figure 3F). The decline in the number of labeled cells could be fit with an exponential decay curve with a half-life of 11 days (R^2^ = 0.92). Our data also could be fit with two phases, suggested sub-populations with half-lives of ≈9 days and >24 days respectively (R^2^ = 0.91). Our current data did not permit a statistical selection between these two models. That is, the goodness of fit for the two models was similar.

### Distinct Longevity for Receptor and Presynaptic Taste Cells

**Figure 4:**
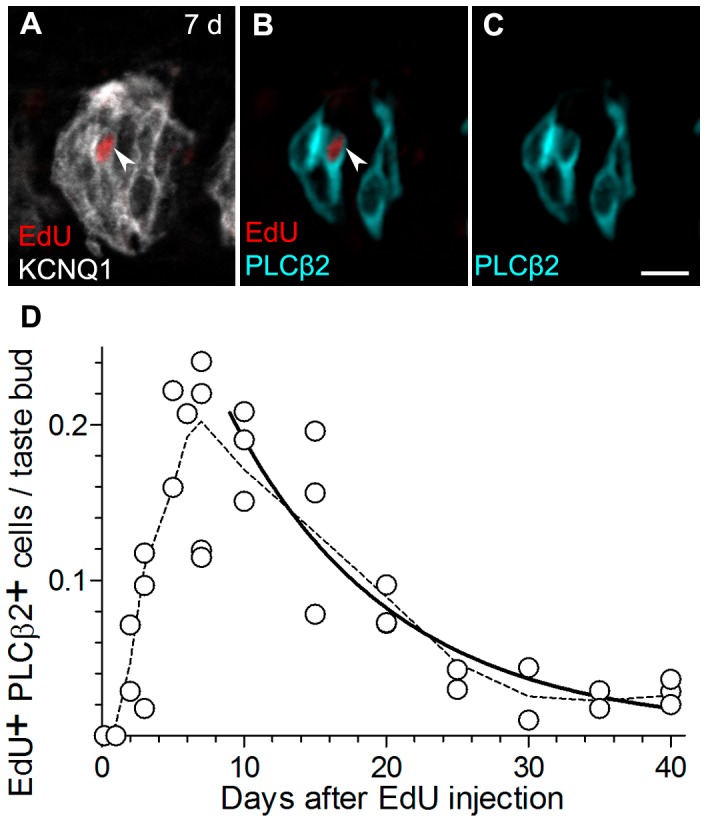
EdU-labeled nuclei are present in Type II cells 2 days post-injection and are mostly eliminated by 25 days. **A,** a representative taste bud from a mouse analyzed 7 days post-injection, viewed for EdU and KCNQ1 immunofluorescence as above. One EdU-labeled nucleus (arrowhead) is completely embedded in the KCNQ1-stained taste bud. **B,C,** the same nucleus (arrowhead) is seen to be contained in a PLCβ2+ cell. **D,** Aggregate data from 35 mice for EdU+ nuclei located in PLCβ2+ cells. As in Figures 2,3, each symbol represents data from one mouse. The solid line is a 1-phase exponential decay curve. The average half-life of EdU+ PLCβ2+ cells is calculated to be 8 days (R^2^ = 0.84). The dashed line is a smoothed line through averages at each time point. Scale bar, 20 µm.

To assess more confidently whether different taste bud cells turn over at different rates, we analyzed EdU incorporation in two distinct classes of taste cells – Type II (Receptor) and Type III (Presynaptic) cells. Type II cells were identified as GFP-positive cells in circumvallate papillae from *PLCb2*-GFP mice (Figure 4A–C). As described above, we also immunostained for KCNQ1 to define the boundaries of taste buds. EdU-labeled Type II cell nuclei first appeared 2 days after EdU injection, and reached a peak at ≈7–10 days (Figure 4D). The decline in EdU-labeled Type II cells could be fit to an exponential decay that yielded a half-life of 8 days (R^2^ = 0.84).

**Figure 5:**
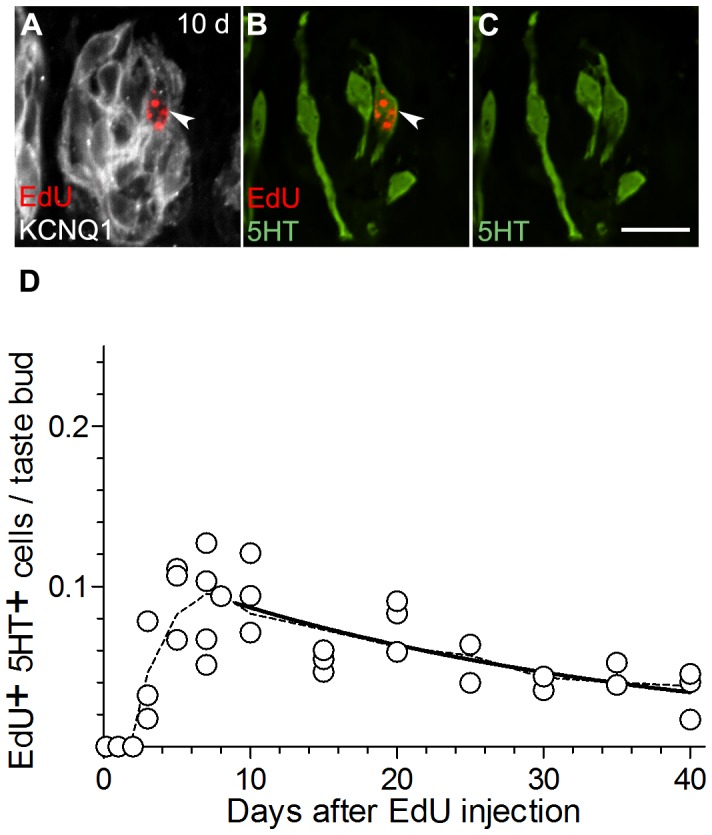
Type III cells become EdU labeled only after 3 days and they persist beyond 40 days. **A, B, C,** a representative taste bud from a mouse 10 days after i.p. injection, stained for EdU (red), KCNQ1 (grey) and 5HT (green). **D,** Aggregate data on the incidence of EdU+5HT+ cells in circumvallate taste buds from 35 mice. The solid line is a non-linear decay curve that yields a half-life of 22 days (R^2^ = 0.54) for Type III cells. The dashed line is a smoothed curve through the mean values at each time point. Scale bar, 20 µm.

Type III taste cells, identified as those staining for 5HT (Figure 5A–C), first showed EdU-labeled nuclei 3 days after EdU injection, and reached a maximum with a broad peak 7–10 days post-injection (Figure 5D). Type III cells are relatively less common than Type II cells, and we noticed considerably more variability in the incidence of EdU+, 5HT+ taste cells from animal to animal. Nevertheless, we were able to fit the data with an exponential decay curve. The half-life of EdU-labeled 5HT+ cells was 22 days (R^2^ = 0.54). We noted that while the large majority of taste buds contained no EdU+, 5HT+ cells, a few taste buds contained 2 or even 3 such nuclei. This suggests the possibility that Type III cells may be born in clusters.

The longer half-life of Type III cells relative to Type II cells (22 days *vs.* 8 days) suggests slower turnover, which in turn predicts that 5HT+ Type III cells should acquire EdU-label less frequently. Indeed, at the peak of labeling (7 d post-injection), >2% of all PLCβ2 cells were EdU-labeled, while <1% of 5HT cells were EdU-labeled. This is consistent with the more rapid entry into and exit of EdU from the Type II cell pool.

If the bio-availability of EdU is similar to that of BrdU, the injected nucleotide is fully incorporated into DNA within the first 4 h [22]. We estimate that a single pulse of EdU is available for incorporation during at most 2.0% of the lifetime of the typical Type II cell (4 h/(8×24 h)) but only 0.8% of the life of the typical Type III cell (4 h/(22×24 h)). If the efficiency of EdU incorporation is similar between the two cell populations, a single 4 h pulse of EdU should then label ≈2.0% of Type II cells and ≈0.8% of Type III cells. Circumvallate taste buds contain 10–12 Type II cells and 6–7 Type III cells as previously reported [26] and as we have confirmed here. Thus, the half-life of each cell type predicts 0.23 EdU-labeled Type II cells (11 cells×2.0%) and 0.05 EdU-labeled Type III cells (7 cells×0.8%) respectively per taste bud. These predicted values are remarkably close to the peak values of 0.21 Type II cells and 0.09 Type III cells per taste bud that we actually observed (Figures 4, 5).

### EdU-labeled Type I and Undifferentiated Cells

Type I (Glial-like) taste cells are the most prevalent class of cells in taste buds [27]. To date, the only marker that confidently identifies most or all these cells is the ecto-ATPase, NTPDase2 [3]. In preliminary experiments, we sought to determine the incidence of EdU-labeled nuclei in NTPDase2+ cells. However, NTPDase2 immunostaining is limited to the plasma membrane of Type I cells. Given the narrow, elongate shape and thin lamellar processes of Type I cells, it is nearly impossible to reliably assign an EdU-labeled nucleus as belonging with plasma membrane staining for NTPDase2. In short, NTPDase2 could not be used to analyze EdU-labeled Type I cells.

**Figure 6:**
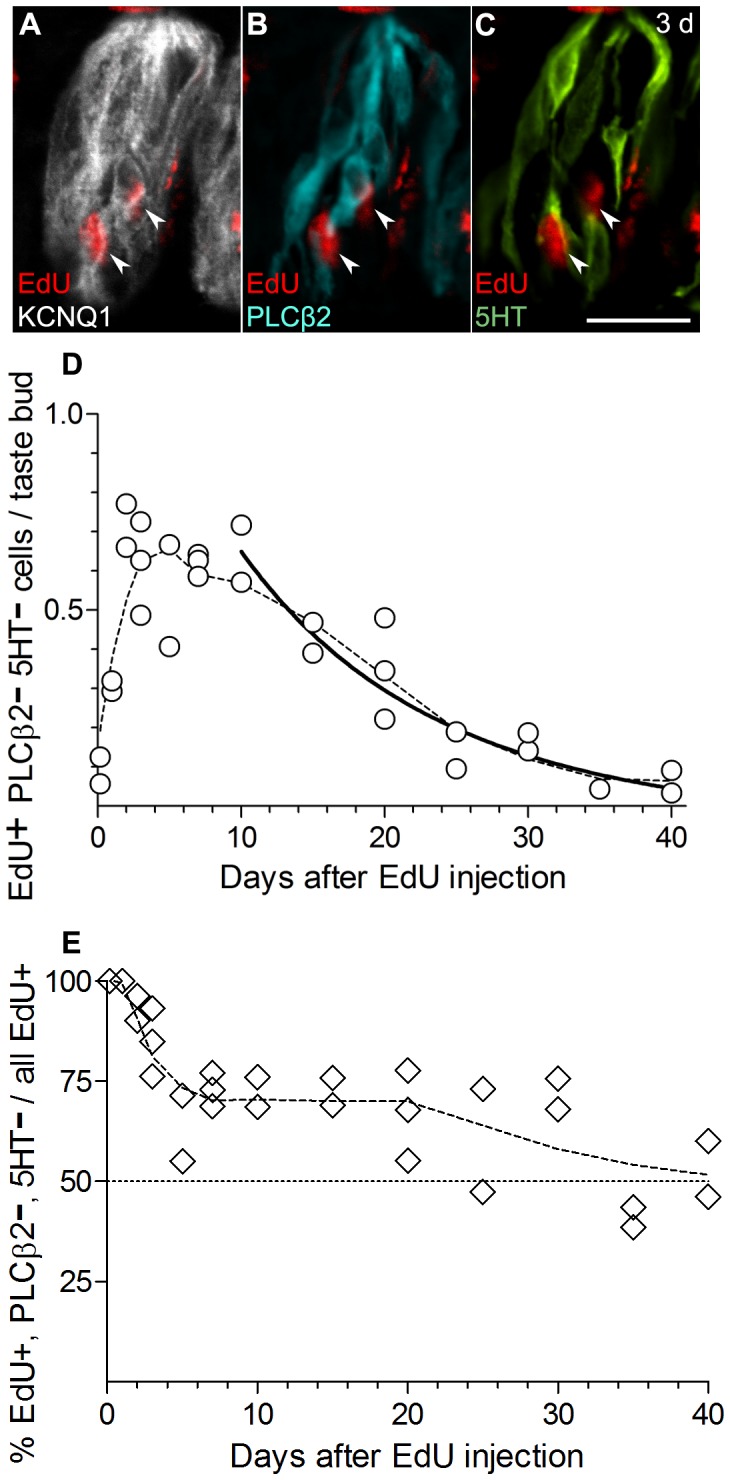
EdU-labeled non-Type II, non-Type III cells enter the taste bud rapidly and constitute the largest fraction of EdU+ cells. This heterogeneous group of cells is defined here simply by the absence of both PLCβ2 and 5HT. **A, B, C,** a taste bud from a mouse 3 days post-injection displays several EdU-labeled cells (arrowheads) of this class. EdU, KCNQ1, PLCβ2 and 5HT were visualized as in previous figures. The indicated EdU+ nuclei do not map onto immunoreactivity for either cell-type marker; but they are clearly within the taste bud perimeter. **D,** Aggregate data from 30 mice for EdU+, PLCβ2^–^, 5HT^–^ cells, fit with a two-phase exponential decay curve. The faster component comprises 80% of the population and exhibits a half-life of 8 days while the slower component contains 20% of the EdU+,PLCβ2^–^,5HT^–^ cells and disappears with a half-life of 24 days (R^2^ = 0.97). The exponential fit was ambiguous insofar as the relative fraction of the two sub-populations could not be discerned precisely. Equivalent goodness-of-fit was obtained for the fast component constituting 60–80% of the cellular population. The EdU+,PLCβ2^–^,5HT^–^ cells are obviously a heterogeneous group as discussed in the text. Different constitutent cell types (e.g. mature Type I cells, immature taste cells, quiescent undifferentiated cells, and perhaps some progenitor cells) may all have dramatically different lifetimes. E, Incidence of these EdU+ PLCβ2^–^ 5HT^–^ cells among all EdU+ cells within the taste buds. As with other graphs, each symbol represents data from a separate mouse. Scale bar, 20 µm.

Hence, we scored EdU-labeled cells that were simply immuno-negative for both PLCβ2 and 5HT (Figure 6A–D). The large majority of such EdU+, PLCβ2, 5HT^–^ cells are either mature Type I cells or immature/undifferentiated taste cells. The set may also include any taste cell types that are as yet undefined (i.e. beyond known Types I, II and III) and to a limited extent, any false negative, i.e. unstained, Type II and III cells.

The number of EdU+,PLCβ2^–^,5HT^–^ cells increased as much as 7-fold over the first 4 days post-labeling (Figure 6D), principally reflecting immigration of newly born cells from the surrounding epithelium. After 10 days post-injection, the population of EdU+,PLCβ2^―^,5HT^―^ diminished with complex dynamics that were best fit with a 2-phase exponential decay curve. Approximatly 60–80% of the population exhibited a half-life of 8 days while the remaining 20–40% decayed with a half-life of 24 days (R^2^ = 0.97).

### EdU-labeled Cell Pairs

**Figure 7:**
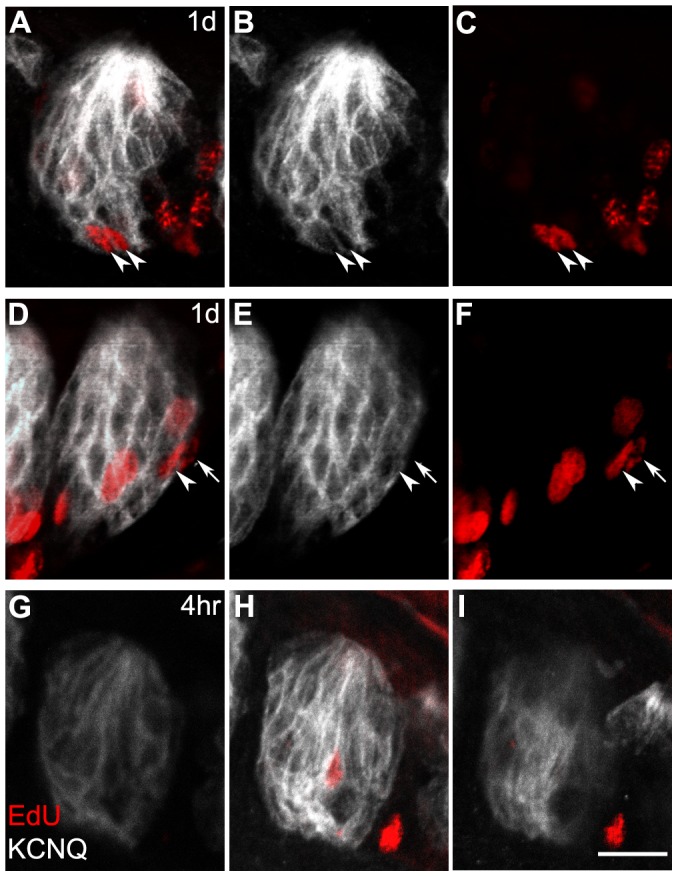
Paired “sister” cells are seen in and around taste buds. A–C, a pair of cells inside and near the base of a circumvallate taste bud, 1 day after EdU injection. Note that in this single plane confocal micrograph, KCNQ1 staining surrounds each of the two labeled nuclei. **D–F,** A pair of sister cells with one cell (arrow) still on the outside of the taste bud; the other cell (arrowhead) appears to be transitioning into the KCNQ1-positive area of the taste bud. These images are from a single optical plane micrograph from a mouse 1 day after EdU injection. **G-I,** An example of an EdU-labeled cell located centrally and away from the basement membrane region. The three panels are consecutive optical sections, each 4–5 µm thick, of a single taste bud.from a mouse 4 h after EdU injection. The appearance of the same taste bud at planes above and below the plane of the labeled cell demonstrates this cell was mid-way through the taste bud in the Z-dimension. Scale bar, 20 µm.

During the course of counting EdU+ nuclei, we noticed pairs of nuclei that may be interpreted as “sister cells”, i.e. the recent products of a mitosis (Figure 7A–F). This interpretation is based on the parallel arrangement, similar shape and size and similar intensity of EdU fluorescence in the two cells of each pair. Such pairs were only observed 1 day after EdU injection. We observed several instances (11 pairs across 3 mice) with both cells of a putative pair included inside the taste bud (arrowheads, Figure 7A–C). In several additional instances (6 pairs observed, one shown in Figure 7D–F), one cell of the pair (arrowhead) was enclosed within the boundary of the taste bud (as evidenced by KCNQ1 staining) while the other cell (arrow) remained outside the taste bud, without a KCNQ1-stained membrane beyond it. This latter pattern is suggestive of an asymmetric mitosis in the basal epithelium with one of the progeny cells entering the taste bud. Alternatively, it may reflect delayed migration of one of the two sister cells. None of the 34 paired cells displayed immunostaining for either mature cell marker (PLCβ2 or 5HT), consistent with their recent birth less than 24 h earlier.

## Discussion

We have used a new method, EdU-labeling, to re-evaluate the birth and turnover of circumvallate taste cells in adult mice. When EdU was injected into adult mice, the nucleotide analog was taken up into proliferating epithelial cells surrounding taste buds. A small but finite number of cells within taste buds also were labeled at the earliest times examined, 4 h post-injection. From 4 h to 7 days post-injection, there is a nearly 10-fold increase in the number of EdU-labeled cells in taste buds. The very large majority of new cells in taste buds are born in the adjacent basal epithelium and migrate into taste buds. By combining EdU-labeling with immunostaining for taste cell-type selective markers, we tracked the lifespan of different types of cells in mouse circumvallate taste buds. In our analysis, Type II cells have a half-life of 8 days. Type III cells appear to survive much longer (22 days on average). Our estimated half-life of 11 days for taste buds as a whole, and of 8 days for Type II cells are consistent with earlier published estimates using other methods [11], [28].

In order to measure the lifetimes of taste cells of each type, it was necessary to select markers that are definitively diagnostic of separate cell classes, and that can reliably be combined for multiple simultaneous immunofluorescence labeling. The taste cell types were originally defined by electron microscopic criteria of cell shape and cytoplasmic density [1], [27]. Subsequent combinations of electron and light microscopic immunohistochemical analyses rendered the classification scheme more broadly usable. In this fashion, 5-HT was shown to be consistently localized to Type III cells with their narrow, spindle shape, elongate nuclei and defined chemical synapses [9], [10]. And PLCβ2, along with other components of the taste transduction machinery, was localized to Type II cells [7]. In the present study, the practicalities of detecting multiple antigens led us to use PLCβ2-GFP mice which we previously showed [18] faithfully express GFP in the appropriate Type II cells. Numerous subsequent reports with coordinated functional and gene expression analyses have substantiated the equivalence of Type II cells with cells that detect sweet, bitter and umami tastants via G protein coupled receptors, and Type III cells with those that respond to sour tastants and secrete several transmitters [2]. Although electron microscopically validated markers exist also for Type I cells, their plasma membrane localization precluded our using them to assign EdU-labeled nuclei to mature Type I cells.

During the early stages of this project, we examined sections from both fungiform and circumvallate taste buds. In both fields, we noticed large variability in the absolute numbers of EdU-labeled cells per taste bud, even when comparing adjacent buds in a single animal. Thus, an accurate estimate of incorporation and turnover requires counting large numbers of taste buds from each mouse. Hence, the present quantitative analysis is focused on circumvallate taste buds. Nevertheless, the results we obtained from fungiform and circumvallate taste buds were qualitatively similar (data not shown). In each case, EdU was incorporated into basal epithelium, cells appear to migrate into taste buds over several days and differentiate into taste cells. Because there are relatively fewer Type III cells per taste bud in the anterior tongue, measuring their lifetimes may be challenging.

The turnover of cells in mammalian taste buds was analyzed using ^3^H-thymidine several decades ago [11], [12], [16]. As in our present findings, ^3^H-thymidine overwhelmingly was incorporated into basal epithelial cells immediately surrounding taste buds. Labeled cells were postulated to migrate into the bud and then have an average lifespan of ≈10 days. A more recent study [17] that employed BrdU-labeling suggested that taste buds contain short- and long-lived cells, with short-lived cells turning over every 2 days. Because Hamamichi et al. [17] did not precisely delimit the boundary of taste buds by confocal microscopy, we believe that the short-lived cell population they reported is non-taste cells immediately *surrounding* taste buds. Indeed, their short-lived cell population was reported to have a lifespan of 2 days, similar to our calculated half-life of 2 days for *extra*-gemmal non-taste epithelial cells that do not enter taste buds (Figure 2F). Similarly, Keratin14+, BrdU+ cells that were reported as progenitors residing within taste buds [29] likely represented proliferative cells immediately *outside* taste buds. Using a marker that precisely delineates taste buds (e.g. Figures 2B, 3A, 7A–I), we show that newly born basal epithelial cells are often closely apposed to the perimeter of taste buds. Without electron microscopy or confocal optical thin-sectioning, it is nearly impossible to distinguish whether these cells are in the interior of a taste bud. Indeed, other studies [30] using confocal microscopy and precisely delimited taste buds correctly indicated the *extra*-gemmal location of cycling cells that produce taste buds.

The literature now seems clear that taste buds are replenished from proliferating cells in the surrounding epithelium. In addition to this extrinsic source for most taste bud cells, are some cells also born inside taste buds? Mitotic figures have occasionally been reported within the boundaries of taste buds [31], [32]. Further, Sullivan et al. [33] reported that taste buds in adult mice contain occasional mitotically active cells, identified as BrdU+ and expressing p63, a transcription factor characteristic of cycling epithelial cells. At our earliest post-injection time point, we too saw a small number of EdU-labeled cells *inside* taste buds (Figures 3F and 7G–I). Although Sullivan et al. [33] had a much smaller sample size than ours, their reported incidence of labeled cells within the boundaries of taste buds, based on confocal imaging, is remarkably similar to ours (Figure 3F). Because the duration of S and (G2+ ½M) phases in mouse lingual epithelium were estimated at 5.3 h and 4.2 h respectively [23], it appears unlikely that cells could have incorporated detectable levels of EdU and completed mitosis within 4 h, our shortest post-injection interval. Thus, we suggest that a small fraction (≤10%) of taste cells may be born inside taste buds, while the very large majority migrate in from the surrounding epithelium. Curiously, ≈half of these *intra-*gemmal newly labeled nuclei were well away from both the basement membrane and the lateral margins of the taste bud (Figure 7G–I). The location of these infrequent, mitotically active cells in our studies and those of Sullivan et al. [33] is reminiscent of the ”glial-like” intermediate progenitor cells that support neurogenesis in the adult brain [34]. If progenitors indeed reside in taste buds, it will be important to determine if their progeny represent a single lineage or give rise to multiple taste cell types. It is tempting to speculate that the infrequently cycling cells within taste buds may give rise to the long-lived Type III cells. We note that we counted ≈0.091 EdU+ cells *inside* taste buds at 4 h (i.e. post-labeling but before cell division), strikingly close to the peak value of ≈0.096 EdU-labeled 5HT+ cells (Figure 5D). Also, at 40 days post-injection, when the longest-lived cell type predominates, we counted ≈0.113 EdU+ cells/taste bud. This concordance supports our notion that Type III cells, unlike other taste cell types, may be born inside taste buds. Indeed, lineage-tracing studies on developing and adult taste buds have suggested that Type III cells are derived from an as yet undefined, progenitor pool separate from progenitors that give rise to taste cell types I and II [35], [36].

Our population of PLCβ2^–^, 5HT^–^ cells must include Type I (glial-like) cells as well as undifferentiated and immature cells. The best fit for our data suggests two sub-populations with very different longevities. Roughly three-fourths of these cells may die-off with a half-life of 8 days, while a quarter may linger in the taste bud with a half-life of 24 days. A possible interpretation of these findings is that the shorter-lived set comprises mature Type I (i.e. NTPDase2+) cells while quiescent cells that gradually differentiate have a longer total life. There are intriguing parallels that can be drawn between our data and earlier reports. Farbman [16], using ^3^H-thymidine incorporation and autoradiography at the ultrastructural level, found that on average, “dark cells” (now recognized as Type I cells [3], [4]) survive for only 7 days while “light cells” (combination of Types II and III [10]) survive longer (although life-times were not estimated). A similar time-frame for dark cell longevity was also observed by Delay et al. [32]. Thus, the major sub-population (60–80%) of EdU+,PLCβ2^―^,5HT^―^ cells we observe, with 8-day half-life may reasonably be considered Type I cells. The minor sub-population (20–40%), with a half-life of 24 days, likely represents immature and undifferentiated taste cells, as discussed below.

Because non-taste epithelium adjacent to taste buds became essentially devoid of EdU-labeled cells by 10 days post-injection (Figure 2F), our results suggest that cells enter taste buds within a few days of their birth. Such newly born cells may remain quiescent within the taste bud for several more days before committing to a differentiated fate. Cells lacking both Type II and Type III markers constituted nearly two-thirds of *all* EdU-labeled taste cells, even up to 30 days post-injection (Figure 6F). If our hypothesis regarding the longer-lived sub-population of EdU+,PLCβ2^–^,5HT^–^ cells is correct, then quiescent and immature cells must represent a significant population of cells in the taste bud. Our data cannot distinguish exactly how numerous are such immature taste cells, nor how long they remain quiescent. The triggers that induce particular EdU+,PLCβ2^–^,5HT^–^ cells to differentiate, and whether they produce one or more different mature cell type are open questions. Small numbers of immature taste cells were previously recognized to be responsive to Wnt signals during their post-mitotic differentiation [37]. Our experiments did not directly address the question of the lineage of each cell type. However, we note that EdU-labeled cells differentiate into PLCβ2+ Type II cells and into 5HT+ Type III, 2 and 3 days respectively after the EdU pulse. This makes it highly unlikely that taste cells sequentially are transformed between Type III and Type II cells as previously suggested [32], [38].

Several recent light microscopic studies in mammals, have invoked the presence of “Type IV” cells in taste buds (e.g. [21], [39], [40]). As discussed above, there are at best, a few stem-like or progenitor cells inside the bud (i.e. cycling cells whose progeny subsequently differentiate). Attempts to characterize progenitor cells using fluorescently labeled antibodies or lectins have been fraught with the problem of accurately identifying their location within the taste bud. Thus, Jacalin and Peanut Agglutinin, which also label basal epithelial cells *between* taste buds, are unlikely to be markers of taste bud-resident progenitors as proposed [39]. Our data and earlier findings [37] indicate that taste buds contain many recently born post-mitotic *undifferentiated* cells. These include both quiescent precursors and immature taste cells; they are not taste *progenitors* (which by definition, are cycling cells). Immature cells are not a homogeneous “cell type”, and indeed, may be on the path to diverse phenotypes. Thus, “Type IV cell” is an imprecise and ambiguously used nomenclature, not parallel to the well defined cell Types I, II and III. We recommend that this term should be abandoned.
